# Opium as a carcinogen: A systematic review and meta-analysis

**DOI:** 10.1016/j.eclinm.2021.100768

**Published:** 2021-02-24

**Authors:** Mohammad Zamiri Bidary, Mehrdad Sahranavard, Arash Akhavan Rezayat, Alireza Omranzadeh, Seyyed Hasan Hoseiny, Ali Kabirian, Amirhossein Sahebkar

**Affiliations:** a Student Research Committee, Faculty of Medicine, Mashhad University of Medical Sciences, Mashhad, Iran; b Health Policy Research Center, Shiraz University of Medical Sciences, Shiraz, Iran; c Universal Scientific Education and Research Network (USERN), Tehran, Iran; d Student Research Committee, School of Pharmacy, Mashhad University of Medical Sciences, Mashhad, Iran; e Biotechnology Research Center, Pharmaceutical Technology Institute, Mashhad University of Medical Sciences, Mashhad, Iran; f Applied Biomedical Research Center, Mashhad University of Medical Sciences, Mashhad, Iran; g School of Pharmacy, Mashhad University of Medical Sciences, Mashhad, Iran

## Abstract

**Background:**

Opium and its pyrolysates have been investigated as potential carcinogenic material through several studies in different body systems; however, the results were controversial and no consensus was achieved with this regard. Thus, we aimed to systematically review and meta-analyze all existed evidence regarding association between opium consumption and cancer.

**Methods:**

Four major electronic databases including ISI Web of Science, PubMed, Scopus, and Embase along with Magiran and SID were searched thoroughly for all published articles from inception up to September 25, 2020. All studies were appraised critically by Newcastle Ottawa Scale (NOS) checklist. Relevant demographic data and the intended results of the selected studies were extracted and their Odds ratios (OR) were pooled using Comprehensive Meta-analysis (CMA). The cumulative risk of opium for developing different cancers was calculated.

**Findings:**

34 studies comprised of 18,230 individuals were entered in our systematic review and finally 32 publications were enrolled in meta-analysis. Overall, using the random effects model, opium consumption was associated with increased rate of malignancies in both minimally[OR = 4.14 95%CI = (3.32–5.15)] and fully adjusted [OR = 4.35 95%CI = (3.36–5.62)] analyses. Moreover, using random effects fully adjusted model, the subgroup analysis revealed increased risk for larynx [OR = 9.58 95%CI = (6.31–14.53)], respiratory [OR = 9.02 95%CI = (6.27–12.96)], head and neck [OR = 8•03 95%CI = (4.03–16.00)], and colon [OR=5.58 95%CI = (3.14–9.92)] cancers for opium consumers compared to non-consumers.

**Interpretation:**

Opium consumption is highly associated with all reported types of cancers, especially in fully adjusted model; however, basic pathophysiology should be further investigated.

**Funding:**

None.

**Research in context**

**Evidence before this research**

Taking into consideration different reports that assessed the carcinogenicity of opium, we tried to pool all available studies in a systematic review and meta-analysis. ISI web of science, Scopus, Embase, PubMed, and Persian databases including Magiran (magiran.com) and SID (sid.ir) were searched for identifying all the studies published until September 25, 2020 without any time or language limitation. The main outcome measure was the association between opium addiction and the risk of cancers. The odds and hazard ratios were pooled in Comprehensive Meta-analysis (CMA) software and the pooled results were reported.

**Added value of this study**

Several studies have reported different odds ratios for the carcinogenic impact of opium use on different cancers but making all these studies together can provide a more robust result. Our findings showed that after adjusting for different confounders, opium use was associated with a 4.35-fold increased risk of malignancy. This addictive substance was associated with a 9.02-fold higher risk of respiratory cancer, 8.03-fold increased risk of head and neck cancer, and 3.03-fold higher risk of gastrointestinal malignancies. In fact, these findings are a summary of 32 different studies and have an added value compared to each individual study.

**Implications of all available evidence**

This study can have several implications. Our meta-analysis gives a final odd ratio for the risk that opium poses on cancer development, and could thus generate awareness if highlighted in the guidelines, online resources, text books and practitioners’ databases. Moreover, with a glance to our final outcomes, the heavy cancerous role of opium can be concluded, even comparable with cigarette smoking. Besides clinicians and healthcare practitioners, policy makers should also be warned about the health burden imposed by opium use to take necessary actions, particularly in countries where opium is widely used.

Alt-text: Unlabelled box

## 1 Introduction

Cancer is the second leading cause of death and is responsible for one out of every six mortality cases, globally [1]. It is the third most common cause of death in Iran [2]. The incidence of cancer is increasing in developing countries due to lifestyle changes such as rises in tobacco consumption, which is the most important risk factor for malignancies and the cause of 22% of cancer-related deaths [1,3]. However, cancer cannot be addressed as a mono-factorial disease or even a multifactorial disorder with completely known risk factors. The disease has a variety of predisposing factors ranging from genetic factors [4] to life style [5], air pollution [6], occupational exposure [7], and even disposal to some addictive materials [8]. Opium may be among these risk factors and controversial studies have been conducted pro [9], [10], [11] and against [12,13] its carcinogenicity.

Opium is a highly addictive substance extracted from the opium poppy and is widely used for recreational purposes, especially in the Middle Eastern countries [14], [15], [16], [17]. According to the World Drug Report, about 29 million people used opiates in 2017, which is 50% higher than previous estimates [18]. Being the neighboring country of Afghanistan, the world's biggest opium producer, Iran, is deeply engaged with opium abuse [19]. Moreover, there is a widely held traditional belief that opium consumption has benefits and improves the cardiovascular system and lowers serum lipids and blood sugar [12,20,21]. Twenty percent of the Iranian population with the age of 15–60 has at least a history of drug abuse, and the most commonly abused drug is Opium [22,23]. Indeed, Iran has the largest number of opium abusers per capita globally, which is 28 out of 1000 in population older than 15 years old [21].

Regular opium abuse is considered a risk factor for developing cancer. Previous studies indicated the association between opium consumption and increased risk of laryngeal [10,24], oral [9], gastric [25,26], esophageal [27], [28], [29], [30], [31], colorectal [32,33], bladder [34], [35], [36], [37], [38], [39], [40], [41], [42], [43], pancreatic [44], [45], [46] and lung [21,47,48] cancer. To the best of our knowledge, there is only one old review study concerned with opium use and different types of cancer; the results were not combined by a meta-analysis, and also some Persian studies were ignored, which probably have affected the results and six years after its publication, the number of related studies has increased by twice. Thus, the results of this previous review could not be used in a pooled assessment and a final risk measurement was not conducted to show the risk that opium poses for each type of cancer. They also, reported that the available evidence at that time was not enough to make a definite conclusion about the carcinogenicity of opium [49].

Taking into account the association between opium consumption and increased risk of cancer, we aimed to perform a systematic review and meta-analysis to evaluate this correlation more precisely with meta-analyses.

## 2 Methods

Our review was designed to answer two questions; 1. Can opium consumption increase the risk of cancer? 2. Which cancers are more associated with opium addiction? The current study has been conducted according to the Preferred Reporting Items for Systematic Review and Meta-Analysis (PRISMA) statement [50]. There is noregistered protocol associated with this study.

### 2.1 Search strategy and selection criteria

A systematic search of several databases such as ISI web of science, Scopus, Embase, PubMed, and Persian databases including Magiran (magiran.com) and SID (sid.ir) was performed in order to identify all studies published from inception until September 25, 2020. No language and time limitations were posed to the study. Moreover, Google scholar was checked using Opium and cancer as key words.

Two independent researchers (M.Z.B. and S.H.H.) performed all searches. A third researcher (M.S.) was also involved as a decision-maker for any disagreements. Also, in order to increase sensitivity of the search, reference list of the relevant studies was hand searched. All articles retrieved from the databases were exported to EndNote (Version X.9). Our final search strategy based on free-text words and controlled vocabulary terms using medical subject headings (MeSH) regarding Opium and cancer was as follows: (“Opium” OR “Papaveretum” OR “Omnopon” OR “Pantopon”) AND (“Neoplasia” OR “Neoplasias” OR “Neoplasm” OR “Tumors” OR “Tumor” OR “Cancer” OR “Cancers” OR “Malignancy” OR “Malignancies” OR “Malignant Neoplasms” OR “Malignant Neoplasm” OR “Neoplasm, Malignant” OR “Neoplasms, Malignant” OR “Benign Neoplasms” OR “Neoplasms, Benign” OR “Benign Neoplasm” OR “Neoplasm, Benign” OR “Carcinoma” OR “Carcinomas” OR "Adenocarcinoma" OR "Adenocarcinomas")

After excluding the duplicate studies, a team of two reviewers (M.Z.B. and S.H.H) independently screened the title and abstract of identified publications for potential eligibility, and any disagreement was settled by the third reviewer (M.S.). Our systematic review included observational studies that investigated the role of opium addiction in cancer development. All case reports, case series, commentaries, letter to editors, published abstracts, unpublished trials, position papers, unstructured papers, dissertations, and animal studies were excluded.

### 2.2 Data extraction and quality assessment

After checking for eligibility, full texts of the included studies were acquired, and the qualified studies underwent full-text review and were read, tagged, and hand-noted by two reviewers (M.Z.B. and S.H.H.). Then, any disagreement was verified by a third reviewer (M.S.). Two independent reviewers (M.Z.B. and S.H.H.) assessed the methodological quality of all studies using the Newcastle-Ottawa scale (NOS) 27, and the third reviewer verified the assessment (M.S.). NOS scores of 1–3 were categorized as low-quality, 4–6 as moderate quality, and 7–10 as high-quality studies [51].

The eligible studies underwent data extraction by two independent authors (M.Z.B. and S.H.H.) using predetermined forms. Data extraction table included the information regarding the first author name, journal name, study location, publication year, study design, number of cases and controls separated by age and sex, and the procedure of selection, inclusion and exclusion criteria, risk ratio, odds ratio and hazard ratio. The main outcome measure was the association between opium addiction and the risk of cancers.

### 2.3 Classification of cancers

The odds ratios were pooled based on different classifications of cancer sites using International Classification of Diseases for Oncology, third version (ICD-O-3) [52], classifications reported by International Agency for Research on Cancer (IARC) Monographs on the Identification of Carcinogenic Hazards to Humans [53], or the U.S. National Cancer Institute's (NCI) Surveillance, Epidemiology and End Results (SEER) Training modules [54].

### 2.4 Data analysis

To assess the association between opium use and cancer development, data from separate studies were pooled. The intended effect sizes were odds ratio (OR) and 95% confidence intervals (CIs). In order to statistically minimize the role of confounders, studies were categorized into two groups: studies that were adjusted for only age and sex (minimally adjusted) and studies that were adjusted for age, sex, and some additional factors (fully adjusted). Furthermore, heterogeneity assessment was conducted using Cochran's Q- test and I^2^ index. A cut-off more than 50% was considered as significant level of heterogeneity. Moreover, a funnel plot was designed for included studies, in order to assess the risk of bias of the studies. All analyses were performed by Comprehensive Meta-Analysis (version 3.3.070, Biostat, Englewood, NJ, USA), and a *p*-value of < 0•05 was considered statistically significant.

### 2.5 Role of the funding source

There is no funding source associated with this study.

## 3 Results

### 3.1 Selection methods

**Fig. 1:**
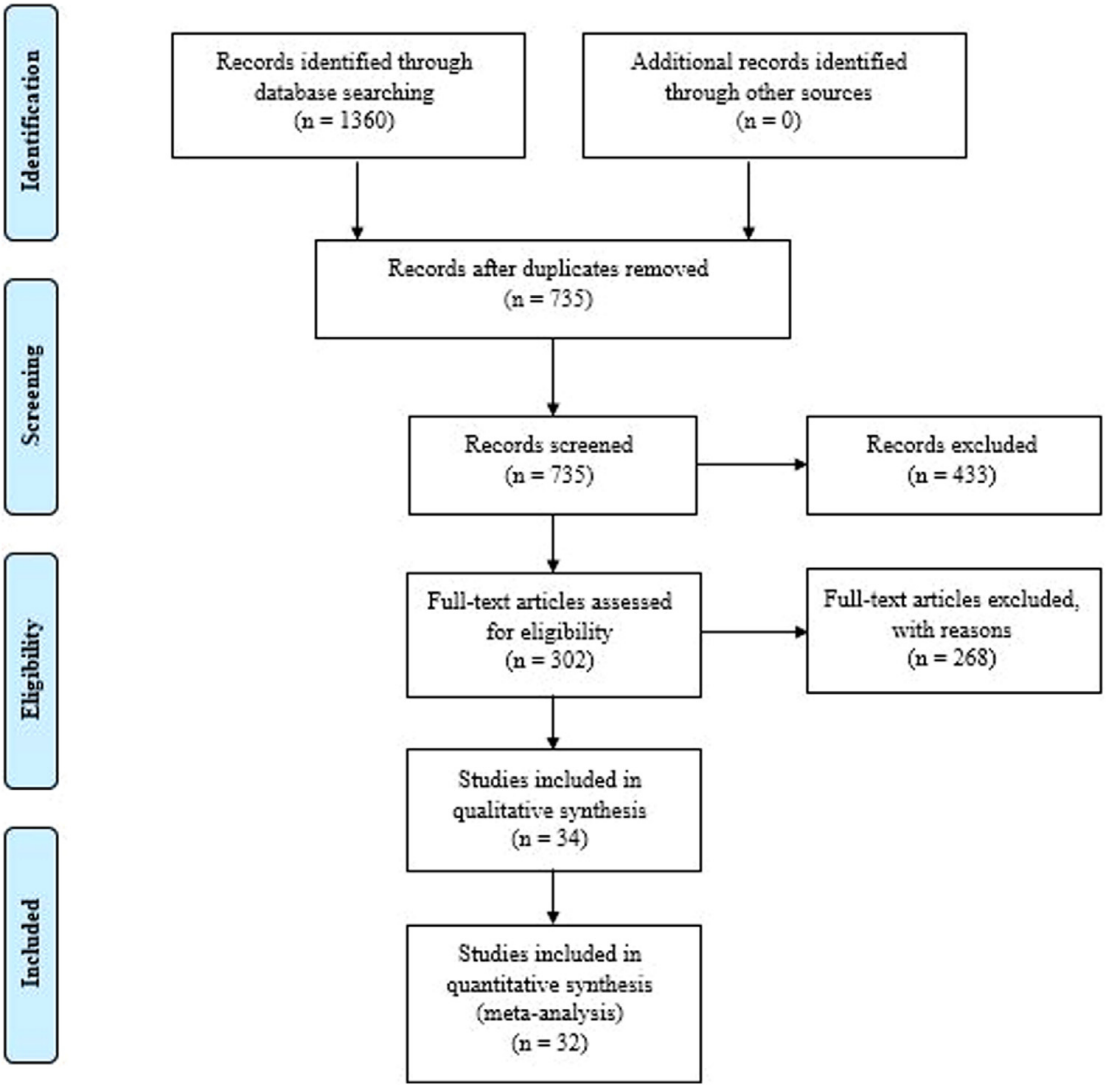
PRISMA flow diagram.

Totally, 244, 256, 305, and 357 studies were identified through performing a comprehensive search in Web of Science, PubMed, Scopus, and Embase databases. As most of our included studies were from Iran, Iranian databases, including SID and MagIran, were also searched for all eligible researches, and 198 papers were included in our primary assessment. No additional relevant studies were entered in our study using Google Scholar and reference screening. Seven hundred thirty-five studies were remained after excluding duplicates and were gone under the title and abstract screening. Therefore, 302 studies were selected for full-text assessment, and finally, 34 and 32 publications were included in our qualitative and quantitative synthesis, respectively. Our study selection procedure is summarized in the PRISMA flowchart (Fig. 1).

### 3.2 Characteristics of the included studies

The detailed data of the included papers such as methodology and population's demographic are summarized in Table 1 and 2. Three cohort [26,33,55] and 31 case-control studies were included in our systematic review. All studies, except one from Singapore [48], were conducted on Iranian people.

**Table 1:** Demographic characteristics of all included studies.

First Author; Pub. Year (Ref.)	Study design	Size	Country (Province/City)	Study period	Inclusion criteria	Exclusion criteria	Matched factors	Opium use definition	QS*
MacLennan, R.; 1977 [48]	Case-Control	533	Singapore (Singapore)	January 1972 to June 1973	Cases: Provisional diagnosis of lung cancer Controls: Hospital inpatients	Following disorders in controls: chronic bronchitis, emphysema, coy pulmonale, myocardial infarction, angina for investigation and cancers of the oral cavity, pharynx, larynx, esophagus, pancreas or bladder	Age; Sex; Dialect	Opium ever smoked	5
Sadeghi, A.; 1979 [43]	Case-Control	198	Iran (Shiraz)	1969 to 1976	Cases: Histopathologically confirmed diagnosis of bladder carcinoma and complete medical history Controls: Inpatients at Nemazee Hospital who had a diagnosis other than cancer, pulmonary disease, or a bladder condition with verified histories regarding opium addiction and cigarette smoking habits	Incomplete histories regarding opium and/or cigarette smoking habits	Age; Sex	–	7
Fahmy, M. S.; 1983 [75]	Case-Control	1381	Iran (Fars)	January 1962 to December 1976	Cases: Histopathologically confirmed diagnosis of oral cavity cancer and complete medical history Controls: -	–	Age, Socioeconomic status	–	6
Toutounchi, M.; 2000 [13]	Case-control	284	Iran (Isfahan)	March 1994 to March 1999	Cases: Histopathologically confirmed diagnosis of bladder cancer Controls: Hospital inpatients in Internal Medicine and Surgery wards except Urology ward; Residence in Isfahan; Ability to answer to interviewers	Controls with history of cancer in other organs or genitourinary system problems	Age (±2 years); Sex	–	7
Mousavi, M. R.; 2003 [76]	Case-Control	410	Iran (Kerman)	September 1996 to September 2002	Cases: Histopathologically confirmed diagnosis of laryngeal squamous cell carcinoma Controls: Patients admitted to otolaryngology department in the study period	Other cancers of the head and neck	Age	DSM-IV criteria for opium dependency and opium consumption for at least 5 years	7
Aliasgari, M. A.; 2004 [42]	Case-Control	160	Iran (Tehran)	1997 to 2000	Cases: Histopathologically confirmed diagnosis of bladder cancer who had undergone surgery Controls: Histopathologically confirmed diagnosis of benign prostatic hyperplasia (BPH) who had undergone surgery	Females	Age, Sex	–	5
Ketabchi, A.; 2005 [41]	Case-Control	242	Iran (Kerman)	1999 to 2003	Cases: Known cases of bladder cancer admitted to urology wards Controls: Tumor-free controls admitted to urology wards	Addiction to other substances except for Opium and having contact with known bladder cancer risk factors (e.g. dye, rubber workers and cigarette smoking)	Age, Sex	Permanent abuse of Opium and its derivatives	7
Nourbakhsh, A.; 2006 [40]	Case-Control	510	Iran (Tehran)	1990 to 2000	Cases: Histopathologically confirmed diagnosis of bladder transitional cell carcinoma (TCC) by pathologic light microscopic examination of the tumor biopsies and having a complete medical record necessary for the study Controls: Admission to trauma ward of Sina Hospital during 1990–2000 without any history, signs or symptoms of urinary problems	–	–	Opium smoking or indigestion in the crude form at least 3 times a week for 5 years or more	7
Nasrollahzadeh, D.; 2008 [31]	Case-Control	871	Iran (eastern Golestan)	December 2003 to June 2007	Cases: Histopathologically confirmed diagnosis of esophageal squamous cell carcinoma (ESCC), being agreed to participate in the study, aged over 18 years old, residing in the study area at the time of registration, and having no history of concurrent cancer in other organs or history of previous cancer in any organ Controls: Population based controls	–	Age (±2 years); Sex; Neighbourhood of residence or village	Opium consumption at least once per week for a minimum of 6 months	6
Hosseini, S. Y; 2010 [39]	Case-Control	358	Iran (Tehran)	March 2004 to March 2008	Cases: Histopathologically confirmed diagnosis of bladder in the urology department Controls: Genetically unrelated healthy subjects without a history of cancer	Previous history of cancer; Metastasized cancer; Previous radiotherapy or chemotherapy, and occupational risk for BC Axis I psychiatric diagnosis, [(DSM)-IV] other than opiate, or caffeine dependence; and use of methadone, levomethadyl acetate, or naltrexone within the 14 days before enrollment.	Age (±5 years); Sex; Geographic origin, Ethnicity; Smoking history	DSM-IV criteria for opium dependency and opium consumption for at least 5 years	6
Shakhssalim, N.; 2010 [38]	Case-Control	1384	Iran (Tehran, Khorasan, Khuzestan, Isfahan and East Azerbaijan)	2006	Cases: Randomly selected newly registered bladder cancer cases at 2006 from regions predicted to have higher incidences Controls: Healthy controls	–	Age (±5 years); Sex; Neighborhood of residence	Previous history of opium consumption	5
Masjedi, M. R.; 2013 [47]	Case-Control	726	Iran (Tehran)	October 2002 to October 2005	Cases: Histopathologically confirmed diagnosis of lung cancer; No suspicion of pulmonary metastases from a different primary tumor; Agreed to undergo a 1•5-hour interview Controls: Inpatients in the same hospital and healthy individuals referred to visit their patients	Control group with neoplasms and respiratory diseases	Age (±3 years); Sex; Place of residence	Consumption of Opium at least once a day for a minimum of 6 months	7
Shakeri, R.; 2013 [25]	Case-Control	922	Iran (Gonbad)	December 2004 to December 2011	Cases: Histopathologically confirmed diagnosis of stomach adenocarcinoma Controls: Healthy individuals selected from Golestan Cohort Study	–	Age; Sex; Place of residence	Opium consumption from more than a year before diagnosis	7
Shokri-Shirvani J.; 2013 [77]	Cross-sectional	961	Iran (Babol)	March 2005 to March 2011	Cases: Endoscopic diagnosis of stomach cancer Controls: All patients who were examined by endoscopy and were not diagnosed as having cancer	History of gastric and esophageal cancer surgery, vagotomy, and gastrojejunostomy; Patients visited for cancer work up	–	Opium consumption for at least 1 year and 3 times a week	7
Hakami, R.; 2014 [28]	Case-Control	120	Iran (Gonbad, Shiraz)	December 2004 to December 2011	Cases: Histopathologically confirmed diagnosis of esophageal squamous cell tumors; Being able to answer the questions; Diagnosed within 6 months prior to the interviews Controls: Controls were recruited from Golestan and Fars provinces (High-risk and low-risk regions, respectively) and they were without any evidence of upper gastrointestinal tract malignancy on endoscopy and they had no family history of esophageal cancer in first-degree relatives	Subjects who had Changed their dietary habits over the past year because of disease or any other reasons	Age (±5 years); Sex	Opium ever used	6
Naghibzadeh Tahami, A.; 2014 [59]	Case-Control	426	Iran (Kerman)	August 2010 to November 2012	Cases: Histopathologically confirmed diagnosis of Upper gastrointestinal (oral cavity, liver, esophagus, stomach, or pancreas) cancers were selected from the northern part of Kerman Controls: Neighborhood controls	Dissatisfaction to participate in the study	Age; Sex; Place of residence	Opium ever used	7
"	"	"	"	"	Cases: Histopathologically confirmed diagnosis of stomach cancers were selected from the northern part of Kerman Controls: Neighborhood controls	"	"	"	"
Razmpa, E.; 2014 [9]	Case-Control	160	Iran (Tehran)	October 2008 to September 2010	Cases: Histopathologically confirmed diagnosis of oral cavity squamous cell carcinoma among 80 consecutive patients who were referred to the ear-nose-throat department Controls: Normal controls	–	Age; Sex; Socioeconomic status	Opium addiction for at least 5 years	7
Sadjadi, A.; 2014 [26]	Cohort	928	Iran (Ardabil)	(A 10-year study)	Cases: Histopathologically confirmed diagnosis of stomach cancer; Age over 40 years' old Controls: -	Participant's refusal; Subject's known gastrointestinal, cardiac or respiratory disease, and pregnancy	–	Opium use for at least once a week for the last 6 months	6
Akbari, M.; 2015 [37]	Case-Control	594	Iran (Shiraz)	2012 to 2013	Cases: Histopathologically confirmed bladder of stomach cancer Controls: Neighborhood controls	–	Age; Sex; Place of residence	Opium ever used	6
Aliramaji, A.; 2015 [36]	Case-Control	350	Iran (Babol)	2001 to 2012	Cases: Histopathologically confirmed diagnosis of bladder cancer who underwent a surgical operation during the study period Controls: Hospital patients referred for assessment of gall bladder stones	Incomplete data	Age; Sex	Opium ever used	7
Ghadimi, T.; 2015 [35]	Case-Control	304	Iran (Kurdistan)	2012 to 2015	Cases: Histopathologically confirmed diagnosis of bladder cancer Controls: Registered patients who referred to the same Clinic/Hospital	For cases: Death and suffering from other cancers; For controls: Having cancer.	Age (±5 years); Sex; Place of residence	–	7
Dianatinasab, M.; 2016 [33]	Cohort	220	Iran (Shiraz)	2009 to 2014	Cases: Diagnosis of colorectal cancer; Having undergone surgery; Not having other cancers in other parts of the body Controls: -	Incomplete data	–	–	6
Lotfi, M. H.; 2016 [78]	Case-Control	400	Iran (Yazd)	2009 to 2013	Cases: Histopathologically confirmed diagnosis of bladder cancer Controls: -	–	Age (±2 years); Sex; Place of residence	Opium ever used	8
Naghibzadeh Tahami, A.; 2016 [32]	Case-Control	525	Iran (Kerman)	January 2012 to December 2014	Cases: Histopathologically and clinically confirmed diagnosis of colorectal cancer Controls: -	–	Age; Sex; Place of residence	Opium ever used	7
"	"	426	"	"	Cases: Histopathologically and clinically confirmed diagnosis of colon cancer Controls: -	"	"	"	"
Shakeri, R.; 2016 [45]	Case-Control	685	Iran (Tehran)	January 2011 to January 2015	Cases: Histopathologically confirmed diagnosis of pancreatic adenocarcinoma Controls: Normal pancreas in the EUS exam; age 40 years or older; a final diagnosis of either asymptomatic small (<10 mm) submucosal lesion in the esophagus or stomach, or a gallbladder or common bile duct stones without cholangitis; no history or current diagnosis of liver failure or renal failure; no history of cancer; no adherence to special diets; no diagnosis of opium-induced common bile duct dilatation or sphincter of Oddi dysfunction; and no development of pancreatic disease or any cancers 1 year after the initial visit.	Not matching with the inclusion criteria	–	Opium use at least weekly for a period of 6 months or more	7
Bakhshaee, M.; 2017 [12]	Case-Control	85	Iran (Mashhad)	September 2008 to August 2010	Cases: Histopathologically confirmed diagnosis of laryngeal cancer Controls: Healthy individuals with no evidence of head and neck or esophageal malignancies	–	Age	Opium consumption at least once a day for a minimum of one year	7
"	"	125	"	"	Cases: Histopathologically confirmed diagnosis of esophageal squamous cell carcinoma Controls: Healthy individuals with no evidence of head and neck or esophageal malignancies	"	"	"	"
Lankarani, K. B.; 2017 [19]	Case-Control	480	Iran (Shiraz)	January 2014 to December 2015	Cases: New histopathologically confirmed Colorectal cancer cases whom registered in the cancer registry system	–	Age (±5 years); Sex; Place of residence	Opium ever used	7
Berjis, N.; 2018 [57]	Case-Control	360	Iran (Isfahan)	2014 to 2015	Cases: Histopathologically confirmed diagnosis of laryngeal squamous cell carcinoma (SCC); Availability of information in patients' records; Possibility of contact with the patient or his family to complete data; Lack of family history of cancer and squamous cell carcinoma of head and neck and other masses except the squamous cell cancer of the larynx Controls: Healthy controls with no laryngeal cancer who were referred to the hospitals which were under investigation	Incomplete data	–	–	5
Pournaghi, S. J.; 2019 [27]	Case-Control	283	Iran (North Khorasan)	2013 to 2015	Cases: Histopathologically confirmed diagnosis of esophageal squamous cell carcinoma (SCC); Age above 18 years; Residence in North Khorasan province; No history of cancer in other organs Controls: Randomly selected hospital inpatients of 2 general hospitals	Inability to answer interviewer's questions; Not consenting to participate in the study	Age; Sex	Opium ever used	6
Vazirinejad, R.; 2020 [58]	Case-Control	285	Iran (Rafsanjan)	2018	Cases: Histopathologically confirmed diagnosis of gastrointestinal cancer in the previous two years Controls: A half of healthy controls were cases' relatives and the others were their neighbors	Non-Iranian people; Consumption of alcohol, nas, and other opioid drugs such as heroin, methadone, and morphine; History of or concurrent cancers	Age; Sex; Place of residence; Smoking	History of opium use more than a year	9
Alizadeh, H.; 2020 [79]	Case-control	420	Iran (Kerman)	January 2014 to December 2017	Cases: Histopathologically confirmed diagnosis of head and neck cancers	–	Age (±5 years); Sex; Place of residence	Opium ever used	6
"	"	"	"	"	Cases: Histopathologically confirmed diagnosis of laryngeal cancer	"	"	"	"
Naghibzadeh Tahami, A.; 2020 [80]	Case-control	420	Iran (Kerman)	January 2014 to December 2017	Cases: Histopathologically confirmed diagnosis of lung cancer	–	Age (±5 years); Sex; Place of residence	Opium ever used	6
Sheikh, M.; 2020 [55]	Cohort	50,034	Iran (eastern Golestan)	January2004 to June 2008	Rural and Urban residents of Golestan Province aged 40–75 years	Previous diagnosis of upper gastrointestinal cancer; Not consent to participate in the study; Temporary residency in the study area	–	Opium ever used	9
Mohebbi, M.; 2020 [81]	Case-control	3698	Iran (10 provinces)	April 2016 to April 2019	Cases: Incident histopathologically confirmed diagnosis of head and neck squamous cell carcinomas Controls: Hospital visitors who visited the hospital for any reason other than receiving treatment	Emergency and maternity wards were excluded for control recruitment	Age; Sex; Place of residence	Opium consumption at least once a week for at least a six-month consecutive period throughout their life	8

⁎ QS: Quality Score from 9.

**Table 2:** Opium consumption and risk of various types of cancers in studies included in the present systematic review.

First Author; Pub. year (Ref)	Cancer (type)	Cases (OU*/ NOU†)	Controls (OU*/ NOU†)	OR‡RR§HR¶	Crude		Minimally Adjusted model			Fully Adjusted model		
					PE||	CI⁎⁎	PE||	CI⁎⁎	Ad factors	PE||	CI⁎⁎	Ad factors
MacLennan, R; 1977 [48]	Lung	233 (66/167)	300 (32/268)	OR	–	–	3•3	2•08–5•26	Age; Sex; Dialect	–	–	–
Sadeghi, A; 1979 [43]	Bladder	99 (45/54)	99 (8/91)	OR	–	–	9•47	4•15–21•60	Age; Sex	–	–	–
"	Bladder	99 (45/54)	99 (8/91)	RR	"	"	2•7	"	Age; Sex	"	"	"
Fahmy, MS; 1983 [75]	Lip and Oral cavity (upper/lower lips, cheek mucosa, gingiva and alveolar redge, floor of the mouth, tongue, and palate)	381 (37/344)	1000 (21/979)	OR	–	–	5•01	2•89–8•68	Age, Socioeconomic status	–	–	–
Toutounchi, M.; 2000 [13]	Bladder	142 (16/126)	142 (7/135)	OR	–	–	2•44	0•97–6•14	Age (±2 years); Sex	–	–	–
Mousavi, MR; 2003 [76]	Larynx (SCC)	98 (23/75)	312 (41/271)	OR	–	–	2•02	1•14–3•58	Age	10•74	5•76–20•02	Age; Sex; Duration of smoking (y); Number of cigarettes per day; Pack-years of smoking; Current smoking status
Aliasgari, M. A.; 2004 [42]	Bladder	52 (13/39)	108 (5/103)	OR	–	–	6•86	2•29–20•53	Age, Sex	–	–	–
Ketabchi, A.; 2005 [41]	Bladder	112 (80/32)	130 (31/99)	OR	–	–	7•99	5•30–12•50	Age, Sex	–	–	–
Nourbakhsh, A.; 2006 [40]	Bladder (TCC)	255 (41/214)	255 (12/243)	OR	3•87	1•98–7•57	–	–	–	–	–	–
Nasrollahzadeh, D.; 2008 [31]	Esophagus (SCC)	300 (90/210)	571 (106/465)	OR	–	–	1•95	1•36–2•78	Age (±2 years); Sex; Place of residence	2	1•39–2•88	Neighborhood of residence or village; Age (±2 years); Sex; Education; Ethnicity
Hosseini, S. Y; 2010 [39]	Bladder	179 (60/119)	179 (7/172)	OR	–	–	12•38	5•47–28•04	Age; Sex	4•6	3•53–6•28	Age; Sex; Cigarette smoking; Family history of cancer
Shakhssalim, N.; 2010 [38]	Bladder (TCC)	692 (67/625)	692 (20/672)	OR	–	–	3•6	2•16–6•00	Age (±5 years); Sex; Place of residence	2•57	1•55–4•26	Age (±5 years); Sex; Neighborhood of residence; Cigarette smoking
Masjedi, M. R.; 2013 [47]	Lung	178 (51/127)	356 (49/307)	OR	–	–	2•51	1•61–3•91	Age (±3 years); Sex; Place of residence	–	–	–
Shakeri, R.; 2013 [25]	Gastric (Adenocarcinoma)	309 (109/200)	613 (131/482)	OR	–	–	2•3	1•6–3•2	Age; Sex; Place of residence	3•1	1•9–5•2	Age; Sex; Place of residence; Ethnicity; Education; Fruit consumption; Vegetable consumption; Socioeconomic status; Cigarette, hookah and nass use
Shokri-Shirvani J.; 2013 [77]	Stomach	281 (18/263)	680 (43/637)	OR	1•01	0•57–1•79	–	–	–	–	–	–
Hakami, R.; 2014 [28]	Esophagus (SCC)	40 (13/27)	80 (9/71)	OR	–	–	3•79	1•45–9•90	Age (±5 years); Sex	–	–	–
Naghibzadeh Tahami, A.; 2014 [59]	Upper Gastrointestinal (UGI) (Oral cavity, stomach, esophagus, liver and pancreas)	142 (54/88)	284 (24/260)	OR	–	–	4•9	2•9–8•4	Age; Sex; Place of residence	4	2•2–7•0	Age; Sex; Place of residence; Specific dietary factors such as consumption of meat, fruit and vegetable, hydrogenated fats, and other key exposure (Smoking)
"	Stomach	89 (34/55)	178 (17/161)	OR	"	"	3•9	2•9–6•8	"	3	1•6–5•6	"
Razmpa, E.; 2014. [9]	Oral cavity	–	–	OR	–	–	4	1•2–13•6	Age; Sex; Socioeconomic status	–	–	–
Sadjadi, A.; 2014 [26]	Stomach	36 (4/32)	892 (14/878)	HR	–	–	4•6	1•6–13•3	Age	3•24	1•37–7•66	Age; Sex; Family history of cancer; Cigarette smoking; Hookah smoking; Alcohol use; Fruit/vegetable intake; Salt intake
Akbari, M.; 2015 [37]	Bladder	198 (43/155)	396 (18/378)	OR	–	–	5•8	3•2–10•5	Age; Sex; Place of residence	3•9	1•3–12•0	Age; Sex; Place of residence; Nutritional factors such as red meat, poultry, fish, hydrogenated oil, olive oil, butter intake, fat intake, fruits, nuts, and moldy food consumption; Alcohol and tobacco use
Aliramaji, A.; 2015 [36]	Bladder	175 (58/117)	175 (27/148)	OR	–	–	2•71	1•62–4•55	Age; Sex	–	–	–
Ghadimi, T.; 2015 [35]	Bladder	152 (16/136)	152 (2/150)	OR	–	–	8•82	1•99–39•08	Age (±5 years); Sex; Place of residence	4•96	1•07–22•92	Age (±5 years); Sex; Place of residence; Smoking history & status; Hypertension; Nephrolithiasis; Radiography; Education; BMI
Dianatinasab, M.; 2016 [33]	Colorectal	220 (16/204)	–	HR	2•49	1•41–4•42	–	–	–	2•8	1•5–4•63	Age; Sex; BMI; Education; Ethnicity; Family history of cancer; Cancer grade; Smoking status; Type of lesion; Occupation; Monthly income
Lotfi, M. H.; 2016 [78]	Bladder	199 (52/147)	200 (21/179)	OR	–	–	3•01	1•73–5•23	Age (±2 years); Sex; Place of residence	–	–	–
Naghibzadeh Tahami, A.; 2016 [32]	Colorectal (Colon, Rectum, and Anus)	175 (45/130)	350 (28/322)	OR	–	–	3•8	2•2–6•6	Age; Sex; Place of residence	4•5	2•4–8•7	Age; Sex; Place of residence; Specific dietary factors such as the use of meat, fruit and vegetables, hydrogenated fats; Cigarette smoking
"	Colon	142 (39/103)	284 (26/258)	OR	"	"	3•7	2•1–6•6	"	5•7	2•7–11•9	"
Shakeri, R.; 2016 [45]	Pancreas (Adenocarcinoma)	357 (57/300)	328 (21/307)	OR	2•77	1•64–4•69	–	–	–	1•91	1•06–3•43	Age; Sex; Place of residence; Alcohol use; Ever use of any type of tobacco
Bakhshaee, M.; 2017 [12]	Larynx	58	27	OR	–	–	9•09	3•21–25•64	Age	6•06	1•10–33•23	Age; Smoking
"	Esophagus (SCC)	98	27	OR	"	"	1•44	0•57–3•62	Age	–	–	–
Lankarani, K. B.; 2017 [19]	Colorectal (Colon, Rectum, and Anus)	160 (32/128)	320 (16/304)	OR	–	–	4•37	2•33–8•22	Age (±5 years); Sex; Place of residence	4•48	2•27–8•82	Age; Sex; Place of residence; Specific dietary factors such as consumption of meat, fruit and vegetable, hydrogenated fats, and other key exposure (Smoking)
"	Colon	93 (18/75)	186 (9/177)	OR	"	"	4•94	2•06–11•88	"	5•4	2•19–13•55	"
Berjis, N.; 2018 [57]	Larynx (SCC)	180 (101/79)	180 (7/173)	OR	31•59	14•04–71•09	–	–	–	18•6	7•9–43•6	Alcohol use; Smoking
Pournaghi, S. J.; 2019 [27]	Esophagus (SCC)	96 (54/42)	187 (76/111)	OR	–	–	1•87	1•14–3•08	Age; Sex	–	–	–
Vazirinejad, R.; 2020 [58]	Gastrointestinal (Esophagus, Gastric, Pancreatic, and Colorectal)	95 (25/70)	190 (12/178)	OR	–	–	–	–	–	5•94	2•37–14•99	Age; Sex; Place of residence; Smoking; Education; Family history of cancer; Consumption of red meat, fruit and vegetables
Alizadeh, H.; 2020 [79]	All Head and Neck cancers (Nasal cavity, paranasal sinuses, oral cavity, pharynx, larynx, and salivary glands cancers)	140 (98/42)	280 (75/205)	OR	–	–	11•82	6•07- 23•0	Age (±5 years); Sex; Place of residence	8•13	4•08- 16•21	Age (±5 years); Sex; Place of residence; Specific dietary factors such as the use of meat, fruit and vegetables, hydrogenated fats, olive oil; Education
"	Larynx	111 (88/23)	222 (64/158)	OR	"	"	17•52	7•56–40•60	"	11•98	5•05–28•39	"
Naghibzadeh Tahami, A.; 2020 [80]	Lung	140 (83/57)	280 (55/225)	OR	–	–	9•73	5•21–18•15	Age (±5 years); Sex; Place of residence	5•95	1•87–18•92	Age (±5 years); Sex; Place of residence; Specific dietary factors such as the use of meat, fruit and vegetables, hydrogenated fats, olive oil; Alcohol consumption; Ciggarette smoking; Education
Sheikh, M.; 2020 [55]	All cancers	1833	Cohort: 50,034	HR	–	–	–	–	–	1•4	1•24–1•58	Sex, ethnicity (Turkman vs non-Turkman), residence (urban vs rural), wealth score quartiles, smoking cigarettes (in the subgroups of tobacco users and entire cohort, fitted as ever vs never), cumulative pack-years of smoked cigarettes, and regular alcohol drinking (ever vs never).
"	Gastrointestinal	914	Cohort: 50,034	HR	"	"	"	"	"	1•31	1•11–1•55	"
"	Respiratory	154	Cohort: 50,034	HR	"	"	"	"	"	2•28	1•58–3•30	"
"	Esophagus	342	Cohort: 50,034	HR	"	"	"	"	"	1•38	1•06–1•80	"
"	Stomach	318	Cohort: 50,034	HR	"	"	"	"	"	1•36	1•03–1•79	"
"	Lung	116	Cohort: 50,034	HR	"	"	"	"	"	2•21	1•44–3•39	"
"	Colon	95	Cohort: 50,034	HR	"	"	"	"	"	0•9	0•48–1•67	"
"	Brain	80	Cohort: 50,034	HR	"	"	"	"	"	1•13	0•61–2•09	"
"	Pancreas	78	Cohort: 50,034	HR	"	"	"	"	"	1•54	0•87–2•72	"
"	Liver	75	Cohort: 50,034	HR	"	"	"	"	"	1•22	0•68–2•18	"
"	Bladder	47	Cohort: 50,034	HR	"	"	"	"	"	2•86	1•47–5.55	"
"	Larynx	38	Cohort: 50,034	HR	"	"	"	"	"	2•53	1•21–5•29	"
Mohebbi, M.; 2020 [81]	Head and Neck SCCs (Lips, oral cavity, pharynx, larynx, and some other sub-sites)	663 (295/368)	3065 (401/2664)	OR	–	–	5•33	4•42–6•41	Age; Sex; Place of residence	3•76	2•96–4•79	Age; Sex; Place of residence; Pack-years of cigarette smoking; Head-years of water-pipe smoking; Regular alcohol drinking; Socioeconomic status, Oral health (DMF index)
"	Lip and Oral cavity	254 (33/221)	3065 (401/2664)	OR	"	"	0•99	0•68–1•45	"	1•53	0•97–2•41	"
"	Pharynx	54 (17/37)	3065 (401/2664)	OR	"	"	3•05	1•70–5•47	"	2•90	1•40–6•02	"
"	Larynx	327 (231/96)	3065 (401/2664)	OR	"	"	15•99	12•32–20•73	"	6•55	4•69–9•13	"

⁎ OU: Opium users;.

† NOU: Non opium users;.

‡ OR: Odds ratio;.

§ RR: Relative risk;.

¶ HR: Hazard ration;.

|| PE: Point estimate;.

⁎⁎ CI: Confidence interval.

### 3.3 Critical appraisal

NOS scores of all of the included studies were between 5 and 9. Medium and high quality studies were included, which were qualified enough to be included in our systematic review and meta-analysis [56]. Four studies scored 5 as the lowest quality [38,42,48,57], and 2 of them scored 9 as the highest quality [55,58]. Table 1 shows the details of NOS scores for each study.

### 3.4 Meta-analysis

**Fig. 2:**
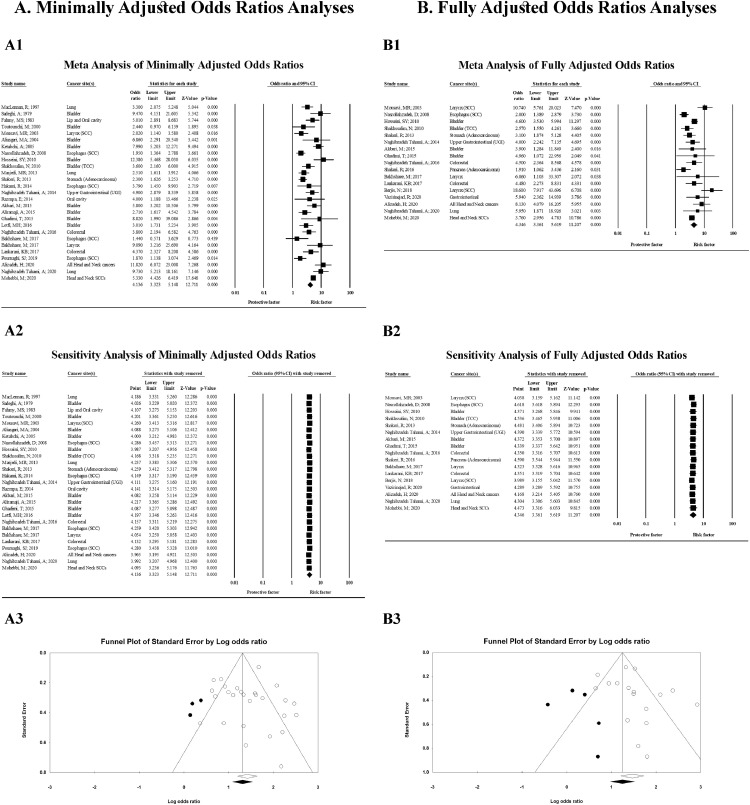
Random effects model meta-analysis of A) Minimally adjusted odds ratios A1. Forest plot; A2. Sensitivity analysis; A3. Funnel plot; B) Fully adjusted odds ratios B1. Forest plot; B2. Sensitivity analysis; B3. Funnel plot; of all cancers regarding opium consumption.

Totally, the odds ratio of 27 studies minimally adjusted method and a population of 15,889 participants were pooled using a random effects analysis, showing a significant association between opium consumption and cancer development (OR = 4•14; 95% CI = 3•32–5•15; *p* < 0•001) (Table 3 and Fig. 2-A1). Similarly, in the random effects model, pooled results of 17 studies with fully adjusted method, including 12,257 participants, showed a significant association between opium use and cancer incidence (OR = 4•35; 95% CI = 3•36–5•62; *p* < 0•001) (Table 3 and Fig. 2-B1). Indeed, we found that the risk of each type of cancer increased significantly with opium consumption. Publication bias assessments for association between opium consumption and cancer development are shown in Fig. 2-A3 and 2-B3, respectively.

**Table 3:** Pooled analysis of Odds Ratios (ORs) regarding the association between opium consumption and cancer.

Cancer type	No. of Studies	Pooled Sample Size	Heterogeneity		Fixed-effects model analysis			Random effects model analysis		
			*p*-value	I[2]	Effect size(95% CI)	Two-tailed test		Effect size(95% CI)	Two-tailed test	
						Z-value	*p*-value		Z-value	*p*-value
Pooled odds ratios of Minimally adjusted studies										
Bladder	10	4274	0•003	64•39	4•94 (4•04–6•05)	15•52	<0•001	5•15 (3•58–7•39)	8•88	<0•001
Larynx	4	4220	<0•001	92•99	11•46 (9•17–14•32)	21•47	<0•001	8•41 (2•73–25•93)	3•71	<0•001
Lung	3	1679	0•002	83•86	3•69 (2•78–4•91)	8•98	<0•001	4•19 (2•03–8•65)	3•87	<0•001
Oral cavity	3	4860	<0•001	91•85	1•76 (1•3–2•38)	3•67	<0•001	2•6 (0•74–9•05)	1•50	0•135
Esophagus	4	1399	0•518	0•00	1•98 (1•52–2•58)	5•02	<0•001	1•98 (1•52–2•58)	5•02	<0•001
Stomach	2	1189	0•023	80•59	3•12 (2•49–3•91)	9•90	<0•001	3•02 (1•80–5•06)	4•19	<0•001
Colon	2	705	0•588	0•00	4•03 (2•5–6•52)	5•70	<0•001	4•03 (2•50–6•52)	5•70	<0•001
Colorectal	2	1005	0•743	0•00	4•04 (2•67–6•11)	6•60	<0•001	4•04 (2•67–6•11)	6•60	<0•001
UGI	6	2588	0•016	64•30	2•58 (2•17–3•06)	10•80	<0•001	2•42 (1•76–3•34)	5•42	<0•001
GI	8	3593	0•012	60•99	2•75 (2•35–3•23)	12•51	<0•001	2•72 (2•06–3•60)	7•06	<0•001
Respiratory	7	5899	<0•001	93•54	7•46 (6•26–8•90)	22•45	<0•001	6•22 (2•90–13•33)	4•70	<0•001
Head and Neck	6	6184	0•003	71•76	5•19 (4•42–6•08)	20•19	<0•001	5•19 (3•32–8•11)	7•23	<0•001
Aerodigestive tract	13	8910	<0.001	82.79	3.95 (3.49–4.47)	21.82	<0.001	3.73 (2.64–5.28)	7.44	<0.001
Overall	27	15,889	<0•001	76•30	4•02 (3•65–4•43)	28•33	<0•001	4•14 (3•32–5•15)	12•71	<0•001
Pooled odds ratios of Fully adjusted studies										
Bladder	4	2640	0•254	26•26	4•07 (3•24–5•11)	12•12	<0•001	3•83 (2•73–5•38)	7•75	<0•001
Larynx	5	4580	0•144	41•63	8•31 (6•4–10•79)	15•89	<0•001	9•58 (6•31–14•53)	10•62	<0•001
Lung	1	420	NA	NA	NA	NA	NA	NA	NA	NA
Oral cavity	1	3319	NA	NA	NA	NA	NA	NA	NA	NA
Esophagus	1	871	NA	NA	NA	NA	NA	NA	NA	NA
Stomach	2	1189	0•936	0•00	3•06 (2•07–4•53)	5•59	<0•001	3•06 (2•07–4•53)	5•59	<0•001
Colon	2	705	0•928	0•00	5•58 (3•14–9•92)	5•86	<0•001	5•58 (3•14–9•92)	5•86	<0•001
Colorectal	2	1005	0•993	0•00	4•49 (2•81–7•16)	6•30	<0•001	4•49 (2•81–7•16)	6•30	<0•001
UGI	3	2060	0•296	17•75	2•44 (1•86–3•18)	6•53	<0•001	2•48 (1•83–3•35)	5•89	<0•001
GI	7	4035	0•072	48•25	2•8 (2•27–3•45)	9•61	<0•001	3•03 (2•23–4•12)	7•08	<0•001
Respiratory	6	5000	0•209	30•15	8•18 (6•34–10•55)	16•16	<0•001	9•02 (6•27–12•96)	11•87	<0•001
Head and Neck	5	5003	<0•001	82•00	4•99 (4•06–6•13)	15•32	<0•001	8•03 (4•03–16•00)	5•92	<0•001
Aerodigestive tract	7	6294	<0.001	85.36	4.04 (3.38–4.82)	15.46	<0.001	6.04 (3.39–10.77)	6.1	<0.001
Overall	17	12,257	<0•001	69•80	3•91 (3•46–4•42)	21•87	<0•001	4•35 (3•36–5•62)	11•21	<0•001

UGI: Upper gastrointestinal; GI: Gastrointestinal.

### 3.5 Sensitivity analysis

To test the power of our finding about association between opium consumption and cancer development in minimally adjusted model, we recalculated the joined consequences of primary investigation by barring one examination. The overall pooled range showed that exclusion of any studies did not change our result (ranged from 3.97 (95% CI = 3.20–4.92) to 4.29 (95% CI = 3.46–5.31)) (Fig. 2-A2). Also, regarding the association between opium consumption and cancer development in fully adjusted odds ratios, the overall pooled range showed that exclusion of any studies did not change our result (ranged from 3.99 (95% CI = 3.16–5.04) to 4.62 (95% CI = 3.62–5.89)) (Fig. 2-B2).

### 3.6 Risk of bias assessment

Fig. 2-A3 presents funnel plot of the included studies assessing the association between total risk of cancer and opium consumption using minimally adjusted odds ratios. The results present an intercept = 0.24, standard error= 0.89, and *p*-value (two-tailed) = 0.789 in Egger's regression intercept that show publication bias does not exist minimally adjusted studies. The results of fully adjusted funnel plot are presented in Fig. 2-B3. The calculated intercept, standard error, and p-value (two-tailed), were 1.10, 0.89, 0.232 in Egger's regression intercept, respectively that show publication bias does not exist in the fully adjusted studies, too.

### 3.7 Subgroup analysis

Random effects model analysis of eight gastrointestinal (GI) cancer studies with 3593 participants and medium heterogeneity (I^2^ = 60.99; *p* = 0.012) was conducted. The results revealed that Opium use was significantly associated with GI cancers (OR = 2.75; 95% CI = 2.35–3.23; *p* < 0.001). Random effect analysis was carried out on six studies with a sample size of 2588 concerning upper GI (UGI) cancers; Thus, a significant association was observed (OR = 2.42; 95% CI = 1.76–3.34; *p* < 0.001). It should be indicated that as ICD-O, IARC, and NCI did not categorized oral cavity cancer in GI or UGI malignancies, we omitted Naghibzadeh Tahami et al. [59] study from our GI and UGI meta-analyses. Likewise, a fixed-effect analysis of four studies, including 1399 participants, showed that esophagus cancer was significantly associated with opium consumption (OR = 1.98; 95% CI = 1.52–2.58; *p* < 0•001; I^2^ = 0•00). Oral cavity cancers were also found to be associated with opium use (OR = 2.6; 95% CI = (0.74–9.05)) in random-effect analysis and minimal adjustment level. Moreover, we detected a significant association between opium use and bladder cancer in both minimally (M) and fully (F) adjusted random effects model (M: OR = 5•15; 95% CI = 3•58–7•39; *p* < 0.001, F: OR = 3.83; 95% CI = (2.73–5.38)). Random effects model analysis of 5 studies with fully adjusted odds ratios and a population of 4580 participants revealed considerably increased odds of laryngeal cancer following opium use (OR = 9•58; 95% CI = 6•31–14•53; *p* < 0.001). In addition to laryngeal cancer, our meta=analyses concluded that respiratory (OR = 9.02 95%CI = (6.27–12.96)), head and neck (OR = 8.03 95%CI = (4.03–16.00)), colon (OR = 5.58 95%CI = (3.14–9.92)) cancers were also significantly associated with the consumption of opium in random-effect fully-adjusted models. Random-effect model also revealed that lung cancer (OR: 4.19; 95% CI = (2.03–8.65)) involvement is also associated with opium consumption. Furthermore, random effects model analysis of minimally adjusted odds ratios showed a significant association between opium use and lung cancer (OR = 4•19; 95% CI = 2.03–8.65; *p* < 0.001). Table 3 and Supplementary Figs. 1–14, indicate the details of all our meta-analyses' results.

## 4 Discussion

Our results were highly suggestive of opium carcinogenicity in different parts of the body. Opium can cause cancer in different parts of the digestive system, from the oral cavity to the other parts of the upper and lower gastrointestinal (GI) system. Our results in fully adjusted odds ratio model showed that opium addicts have around 2•72 times more risk of GI cancers. Moreover, we found that opium consumption makes the person 2•42 times more susceptible to upper GI (UGI) cancers, especially around two times more prone to esophagus cancer and 2•6-fold more prone to oral cavity malignancies. It is reported that morphine, as a dominant alkaloid of Opium, inhibits clearance of *N*-nitrosamines that are proved to be carcinogenic for esophagus cancer. Nitrosamines are usually synthesized in the GI tract through digestion [60].

Moreover, opium and morphine pyrolysates showed mutagenic activity through frameshift mutations in *Salmonella typhimurium* strains [11]. These products cause mutation as sister chromatid exchanges in *S Typhimurium* strains, which is reported to be even at higher rates than those for cigarette condensates [61]. Morphine, as a predominant alkaloid of Opium, also has presented genotoxic ability through DNA methylation [62].

Besides being carcinogenic for digestive tract, opium is found to be responsible for respiratory tract cancers including malignancies of the larynx and the lung. We found that this substance can increase the risk of larynx and lung cancers by 8.41 and 4.19 folds, respectively. Intra-tracheal use of Opium and Morphine pyrolysates has shown to be carcinogenic in hamsters and can cause tracheal carcinoma [63]. Furthermore, the induction of Mu opioid receptors increases opioid-induced malignant growth in lung cancer [64]. Although the exact pathogenesis of Opium on laryngeal cancer is not fully understood, it seems that the mentioned underlying factors such as production of nitrosamines, aromatic hydrocarbons, and hetroheterocyclic compounds produced due to heat exposure of the Opium can be responsible for this carcinogenesis [11,65]. Moreover, additives that may be found in the Opium, such as morphine and codeine, can slow the peristalsis in the smooth muscles of the upper aerodigestive tract and prolong this exposure [49].

Opium is also responsible for bladder cancer. The hypothesis proposed for bladder cancer in the exposure of Opium is similar to those of the other discussed cancer. In fact, the urinary retention caused by Opium can further prolong the exposure to the carcinogenic material [49,66]. We found that after using fully adjusted odds ratios, the risk of bladder cancer was 3.83-fold higher in opium users. A pooled analysis study reported that a person with 40 years of cigarette smoking has a 3.79 higher risk of bladder cancer [67]. This shows that the risk that poses opium consumption on bladder cancer may be even higher than being a forty-year tobacco smoker. However, more studies are needed to conclude this comparison.

Several remnants are also derived from Opium that can be abused. The burned residues of the smoked Opium named *Sukhte* is one of them. *Sukhte* can be boiled in order to produce another substance named *Shireh. Sukhte* is usually ingested, but *Shireh* can be ingested or smoked [68]. These remnants were also reported to be carcinogenic [63]. Many of the studies in the case of the basic pathophysiology of malignant growth with opium consumption mainly focused on opium pyrolysates [11,63], and it is better to study the purified Opium and not its pyrolysates.

Furthermore, the role of the confounders should not be ignored. Many of the opium users usually smoke cigarettes and use alcohol that both of them are carcinogens [8]. However, it is reported through the literature that Opium can be cancerous through both oral and Inhalation pathways in both cigarette smokers and non-smokers [55]. Still, it is advisable for the researchers to try to make these confounders the least and adjust it statistically whenever possible. However, cancer development is a multifactorial problem, and even malnourishment and other lifestyle risk factors should be considered [69].

Drug dealers usually add lead, as an impurity, to the Opium that is also a carcinogen compound and may play a part in this issue [70,71]. Furthermore, the dose and duration of opium consumption should be investigated. We have a pack-year unit for cigarette smoking amount [72]; however, there is no easy measuring method for opium consumption. Some proposed *Nokhod-day* or *Nokhod year* unit, but it still needs more workup [65,73]. It is proposed that each *Nokhod* has an amount of 0•2 mg of Opium [49]. Controlling all these confounders is not possible, and with this regard, animal studies may give better understanding of the role of pure Opium in malignancy development.

Recently, a news published by The Lancet Oncology has reported the work of a group of scientists at International Agency for Research on Cancer (IARC) in which they had performed a thorough review on the opium carcinogenesis. Until now (19^th^ January 2021) and up to our knowledge, this study is currently in press and as we know, they had thoroughly reviewed all studies regarding the carcinogenicity of opium in their Monograph; However, we found that no meta-analyses were done in this review as we done in our study to quantify the potential of opium in development of different cancers [74].

Due to the high heterogeneity regarding the route of opium administration between the included studies, our study was limited to this factor. We tried to minimize the confounding, especially smoking and alcohol consumption, by statistical adjustment, and our results showed a strong relation between Opium and various cancers. What remains little-known is the underlying pathophysiology of carcinogenesis with opium consumption. Future animal and biological studies should further work on the carcinogenesis of Opium. Moreover, small number of the included studies had reported the route of opium administration in their target population and so we recommend future researches to investigate and report it more accurately. In addition, they have to consider that in Islamic countries like Iran, heavy smokers are more vulnerable to break the religious prohibitions and consume alcohol, illegally; Thus, the researchers should keep these factors in mind as they are proven to be strongly associated with cancer development.

We concluded that Opium could be a carcinogen material that can cause various cancers in GI, urinary, and respiratory systems. In fact, we can say that opium consumption can cause different cancers from the oral area to the anus. Yet, the underlying pathophysiology of this carcinogenesis is not fully discovered. More animal studies should be conducted to entirely describe the pathways that lead to carcinogenesis.

## Declaration of Competing Interest

The authors have nothing to disclose.

## Appendix Supplementary materials

Image, application 1
