# Supplementary material for: Opium as a carcinogen: A systematic review and meta-analysis
Source: eClinicalMedicine. 2021 Feb 24;33:100768. doi: 10.1016/j.eclinm.2021.100768 (PMC7921501; doi:10.1016/j.eclinm.2021.100768)
Supplement: Supplementary file 1 [file mmc1.docx]

Supplementary Material

**Supplementary figure 1.** Meta-analysis forest plot of minimally adjusted odds ratios related to the risk of bladder, colon, esophageal, laryngeal, lip and oral cavity, lung, and stomach cancers in opium consumers, compared to non-consumers, separately.

**Supplementary figure 2.** Meta-analysis forest plot of minimally adjusted odds ratios related to the risk of colorectal cancers in opium consumers, compared to non-consumers.

**Supplementary figure 3.** Meta-analysis forest plot of minimally adjusted odds ratios related to the risk of upper gastrointestinal (UGI) cancers in opium consumers, compared to non-consumers.

**Supplementary figure 4.** Meta-analysis forest plot of minimally adjusted odds ratios related to the risk of gastrointestinal (GI) cancers in opium consumers, compared to non-consumers.

 **Supplementary figure 5.** Meta-analysis forest plot of minimally adjusted odds ratios related to the risk of respiratory cancers in opium consumers, compared to non-consumers.

**Supplementary figure 6.** Meta-analysis forest plot of minimally adjusted odds ratios related to the risk of head and neck cancers in opium consumers, compared to non-consumers.

**Supplementary figure 7.** Meta-analysis forest plot of minimally adjusted odds ratios related to the risk of aerodigestive tract cancers in opium consumers, compared to non-consumers.

**Supplementary figure 8.** Meta-analysis forest plot of Fully adjusted odds ratios related to the risk of bladder, colon, laryngeal, and stomach cancers in opium consumers, compared to non-consumers, separately.

**Supplementary figure 9.** Meta-analysis forest plot of Fully adjusted odds ratios related to the risk of colorectal cancers in opium consumers, compared to non-consumers.

**Supplementary figure 10.** Meta-analysis forest plot of Fully adjusted odds ratios related to the risk of upper gastrointestinal (UGI) cancers in opium consumers, compared to non-consumers.

**Supplementary figure 11.** Meta-analysis forest plot of Fully adjusted odds ratios related to the risk of gastrointestinal (GI) cancers in opium consumers, compared to non-consumers.

**Supplementary figure 12.** Meta-analysis forest plot of Fully adjusted odds ratios related to the risk of respiratory cancers in opium consumers, compared to non-consumers.

**Supplementary figure 13.** Meta-analysis forest plot of Fully adjusted odds ratios related to the risk of head and neck cancers in opium consumers, compared to non-consumers.

**Supplementary figure 14.** Meta-analysis forest plot of Fully adjusted odds ratios related to the risk of aerodigestive tract cancers in opium consumers, compared to non-consumers.
